# A Self-Referencing Intensity Based Polymer Optical Fiber Sensor for Liquid Detection

**DOI:** 10.3390/s90806446

**Published:** 2009-08-20

**Authors:** David Sánchez Montero, Carmen Vázquez, Ingo Möllers, Jon Arrúe, Dieter Jäger

**Affiliations:** 1 Departamento de Tecnología Electrónica, Universidad Carlos III de Madrid, Leganés, 28911, Madrid, Spain; E-Mail: cvazquez@ing.uc3m.es (C.V.); 2 ZHO/Optoelektronik, Universität Dusiburg-Essen, 47057 Duisburg, Germany; E-Mails: ingo.moellers@uni-due.de (I.M.); dieter.jaeger@uni-due.de (D.J.); 3 Departamento de Electrónica y Telecomunicaciones, ETSII y IT, Euskal Herriko Unibertsitatea, Bilbao, Spain; E-Mail: jon.arrue@ehu.es (J.A.)

**Keywords:** polymer optical fiber, POF coupler, intensity-based optical sensor, bending losses, liquid level

## Abstract

A novel self-referencing fiber optic intensity sensor based on bending losses of a partially polished polymer optical fiber (POF) coupler is presented. The coupling ratio (K) depends on the external liquid in which the sensor is immersed. It is possible to distinguish between different liquids and to detect their presence. Experimental results for the most usual liquids found in industry, like water and oil, are given. K value increases up to 10% from the nominal value depending on the liquid. Sensor temperature dependence has also been studied for a range from 25 °C (environmental condition) to 50 °C. Any sector requiring liquid level measurements in flammable atmospheres can benefit from this intrinsically safe technology.

## 1. Introduction

Recently, Polymer Optical Fibers (POFs) have been widely used in industry and automotive networks. New technologies and applications like Very high bit-rate Digital Subscriber Line (VDSL), Fiber-to-the Home (FTTH) and Internet Protocol Television (IPTV) require reliable high-speed but low cost networks with simple installation, high security and low space consumption [1]. POF technology could satisfy these requirements [2] and, hence, in-home POF based networks are currently being installed.

Furthermore, the increasing in-home networking feasibility can enhance the automation and integration of sensor applications and systems. Recently, it is shown that one seventh of all fabricated sensors are used for home appliances [3]. This rate will increase with a higher demand on home automation and security applications by supporting higher integration and networking of devices in the home.

For home sensing, there are three different categories of sensors that could be required:

- Environment sensors: air pressure, temperature, wind, humidity,
- Monitoring sensors: health care, movement, lighting/illumination,
- Building sensors: statics, burglary/hold-up alarm, fire, gas and liquid detection, etc.

In the optical sensing field, POFs are also experiencing a big growth because they present numerous advantages such as easier handling (more flexibility) and lower cost compared to glass optical fibers. These are some reasons why new POF sensors have appeared and are still appearing, most of them based on optical power intensity detection.

On the other hand, optical fibers have been used for measuring liquid levels in many forms, some are non-intrusive sensors based on light attenuation while passing tank walls as in [4], but being only useful in transparent tanks. Others used monomode fibers with reduced aperture [5] for short distances or POF fibers for longer distances [6,7], or intrusive large arrays of individual fluid-sensitive transducers [8], or fibers with clad and unclad zones [9]. Transducers for point measurements in control level devices are reported in [10,11]. For increasing the sensor sensitivity, bends [12] plus cladding removal and partially core polished [13,14] can be done on POF fibers. But none of them are self-referencing intensity based sensors; so they need additional elements to avoid influence of undesirable intensity perturbations on the measurements. There is an increasing demand on developing self-referencing intensity based sensor networks in remote operation using optical fibers [15].

In this paper a novel self-referencing intensity-based polymer optical fiber sensor for liquid detection is presented. This sensor itself is a coupler that changes its coupling ratio (*K*) depending on the different refraction indexes around it (provided by different alternative liquids surrounding the sensor). In addition, the sensitive parameter *K* has no dependence of transmission power fluctuations or undesirable power losses at the link or in the sensor network; that is why we named it as a self-referencing sensor.

This paper shows a representative example that demonstrates the capabilities of sensors based on polymer optical fibers in home networks for measuring liquid levels in harsh environments such as oil/petrol tanks or bio-mass boilers to be used in condominiums and buildings. The work includes novel device, theoretical study and experimental results.

## 2. Principle of Operation and Simulations

**Figure 1.**
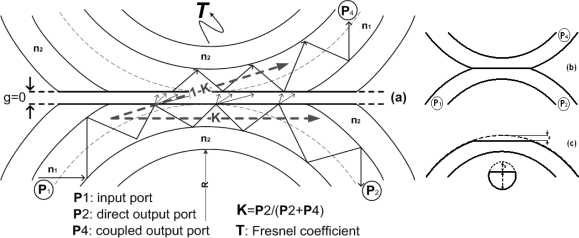
(a) Longitudinal section of the coupling (and sensing) area of the POF liquid sensor. (b) POF liquid sensor. (c) One arm sensor fiber after polishing.

A scheme of the sensor is shown in Figure 1, where P_1_ is the input port and *P_2_* and *P_4_* are, the direct and coupled output ports of the sensor, respectively. The coupling ratio of the device is defined as *K = P_2_/(P_2_ + P_4_). T*, *R* and *g* define, respectively, the Fresnel transmission coefficient, the curvature radius applied at the sensing area and the gap between the cores of the two fibers at the coupling area. Parameter ε indicates the fiber polishing depth.

By introducing a bend on a multimode fiber, the higher order propagating modes in the fiber are refracted because the angle of incidence increases in the interface core-cladding. This refracted light produces an increase of the power losses in the receiver. If the core refractive index, *n_co_*, is approximately equal to the cladding refractive index, *n_cl_*, it can be considered that the Fresnel transmission coefficient *T,* for the beams refracted in an optical fiber, is given by [12]:

(1) $$T=\frac{4\;\text{cos}\;\theta {\left({\text{cos}}^{2}\;\theta -{\text{cos}}^{2}\;\theta_{C}\right)}^{1/2}}{{\left[\text{cos}\;\theta +{\left({\text{cos}}^{2}\;\theta -{\text{cos}}^{2}\;\theta_{C}\right)}^{1/2}\right]}^{2}}$$

where *θ* is the angle of incidence for a certain beam with the normal to the core surface and *θ*_c_ is the critical angle defined as *θ*_c_ = *sin^−1^*(*n_cl_* / *n_co_*). For *θ* ≤ *θ*_c_ the beam will be refracted from the fiber core increasing the power losses. From (1) it can be deducted that for the greater value of *θ*_c_ the greater value of *T* is obtained.

Optical power is transferred and guided in the fiber cladding due not only to the curvature, but also to the polished core (see Figure 1). When the sensor is immersed into different liquids, these losses change because of the different refractive indexes surrounding the coupler which means a variation of the coupling ratio *K* of the sensor and, therefore, a variation of optical power at port *P_4_* at the reception stage.

If the surrounding liquid is oil, its refractive index (*n_oil_* = 1.46) becomes very similar to the refractive index of the fiber cladding (*n_cl_* = 1.417) and all the modes guide through the cladding are refracted outside the fiber. In that case, the value of *T* increases whereas the optical power at port *P_4_* decreases. Consequently, the coupling ratio *K* increases and the coupler becomes more directive. As the liquid refractive index decreases (*n_water_* = 1.33 or *n_air_* ≈ 1) the ratio of the optical power guides again into the fiber core increases as well as the optical power received in port *P_4_*, so *K* decreases. This change of *K* can be measured and, consequently, it is possible not only to distinguish the air-liquid change but also to detect the kind of liquid in which the sensor is immersed.

Use of a coupler as a sensor device intrinsically means a self-referencing sensor. The advantage of this technique is based on the fact that undesirable variations of the optical power at any point of the link between the emitter and the receiver do not change the value of *K*, because it equally affects both sensor outputs (*P_2_* and *P_4_*) and consequently the ratio between them is unaltered. For the same reason it is neither necessary to modulate the source to eliminate the effects of the environmental light which brings considerably cost savings in sensor networks.

Ray tracing method can be used for calculating *T* coefficient in a polished fiber for different bending radius as well as for estimating *K* variations by treating light as rays [12]. In that case, light power can be considered to propagate along the fiber core within infinitesimal cross-section parallel rays and higher radiation losses are obtained due to meridional rays, being those rays contained in a plane defined by the core symmetry axis and the curvature centre of the bend. In order to reduce the computation time of simulations, the fiber cladding-liquid interface can be omitted to a first approach in optical fibers with cladding thickness of 10μm [13] leading to similar results as considering directly fiber core-liquid interface.

**Figure 2.**
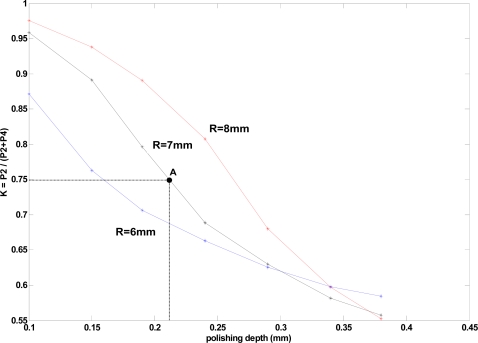
*K* versus polishing depth simulation for different bending radii (6, 7 and 8 mm) considering water as the external media.

Figure 2 shows a simulation of *K* versus polishing depth for different bend radii (*R* = 8 mm, 7 mm and 6 mm) with water as external liquid surrounding the sensor. The aforementioned consideration has been taken into account in the simulation (directly fiber core-liquid interface). The point A corresponds with the fabricated prototype requiring less computational time requirements. Simulations and measurements for that prototype will be discussed later on.

## 3. Sensor Measurements

**Figure 3.**
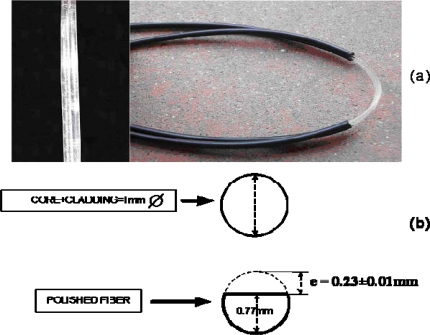
(a) Photograph of the manufactured sensor device and zoom of the sensing area (left side); (b) Schematic of cross section of the fiber core before and after polishing.

For manufacturing the sensor device two standard Step-Index PMMA (polymethylmethacrylate) POFs (*n_co_* = 1.492 and *n_cl_* = 1.417) were used, each one with a section where the cladding was removed and the core was polished (polishing depth was ε = 0.23 ± 0.01mm). Both cores were joined by gluing over the length of the sensing area (3 cm of polished fiber) with no-gap interface (*g* = 0) (see Figure 3).

**Figure 4.**
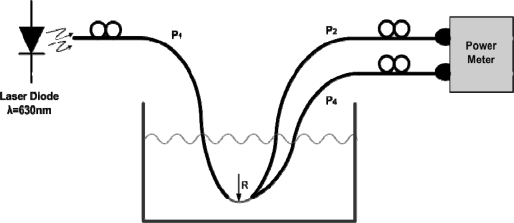
POF sensor set-up for determination of liquid level in tanks.

The set-up scheme for the measurements is shown in Figure 4. A laser diode (Roithner, class III) operating at λ = 630 nm was used to illuminate the POF fiber and a two-input power meter was chosen at the reception stage. The POF sensor was placed in between two POF fibers and was immersed in a tank firstly with water and secondly with oil as the external liquids.

Coupling ratio values for Carthamus tinctorius oil (n = 1.466) and water besides air surrounding the sensor have been experimentally measured having taken five measurements for each sensor configuration (each configuration is determined by the bending radius applied to the sensor and the type of liquid as external media). If we define repeatability as the percentage of the output parameter variation for a determined number of measurement cycles (under the same conditions), the worst case was found to be 1.02% (with R = 25 mm and air as external medium), which was a very good result. All these measurements were taken for an environmental temperature of T = 28.6 °C (average value) with a maximum deviation of ΔT = ±3 °C. Additionally, different bending radii (*R* = 60 mm, 25 mm, 12 mm and 7 mm) at the fiber sensing area were applied and investigated.

**Figure 5.**
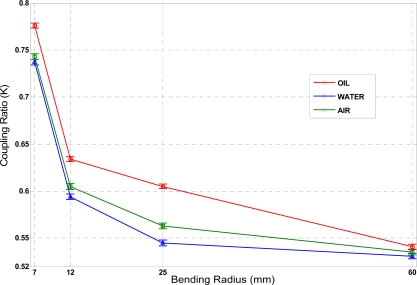
Measurements of the sensor coupling ratio *versus* bending radius, for different liquids. Errors in measurements, given as standard deviation, are also represented for each measurement point.

A self-referencing test was also done to prove the inherent self-referencing property of the proposed liquid level sensor. Figure 5 shows the average value and the measurement errors at each measurement point of the coupling ratio *K* versus different bending radius and different external media. Depending on the media surrounding the sensing area, the value of *K* changed for each fixed bending radius. With proper control electronics and detection schemes the liquid can be distinguished; highest sensitivity was obtained for a bending radius *R* = 25 mm. For this sensor configuration the following coupling ratios were obtained: K_a_ = 0.545 ± 0.0014 (air); K_w_ = 0.563 ± 0.0007 (water) and K_o_ = 0.605 ± 0.0017 (oil) with coupling ratio increments of ΔK = 0.018 (from air to water); ΔK = 0.060 (from air to oil) and ΔK = 0.042 (from water to oil). Measurement errors are given as standard deviation and are, in all cases, at least one order of magnitude below the average value.

In order to make a comparison between measured and simulated results an arbitrary point was selected; obtaining a simulated value of K = 0.75, for ε = 0.23 mm, *R* = 7 mm and water as external medium (see point A in Figure 2). The measured value of the coupling ratio for a manufactured sensor with these parameters was found to have K = 0.744 (see Figure 5). Both the simulated and the experimentally measured *K* values are congruent and show a good agreement between theory and measurements. The simulation technique used at the present paper can help to optimize the design of physic parameters in this type of sensors.

**Figure 6.**
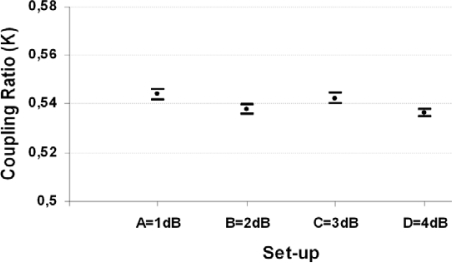
Measurements of coupling ratio of the sensor, for different link losses (up to 4dB from set-up A to set-up D).

The independence of the sensor against hypothetical link losses due to its self-referencing characteristic was also tested as can be seen in Figure 6. From a nominal coupling ratio of K = 0.54 a maximum deviation of ΔK = ±0.002 (six measurements with a temporal space of half and hour between each other were taken per set-up) was obtained. The maximum link losses tested were 4dB and were made by bending the fiber into turns using Mode Scramblers.

## 4. Sensor Temperature Dependence

**Figure 7.**
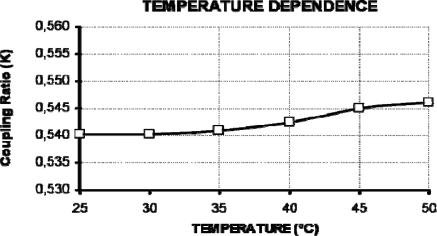
Temperature dependence of the coupling ratio of the sensor (from T = 25 °C to T = 50 °C).

**Figure 8.**
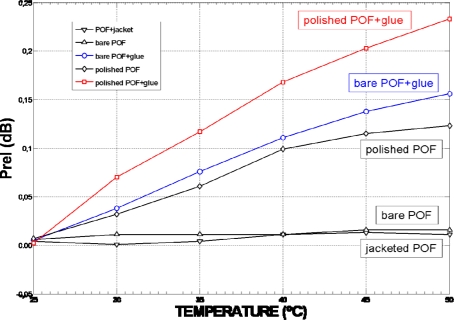
Temperature dependence for different fiber status from T = 25 °C to T = 50 °C.

The temperature dependence of the sensor device was tested inside a climatic chamber over a 25 °C temperature range, from T = 25 °C to T = 50 °C. Figure 7 shows the deviation of *K* at that temperature range. The sensor coupling ratio deviation was found to be around 1% (ΔK ≤ 0.006) from its nominal value. This deviation was mainly produced by the glue used for the sensor manufacturing; showing the low temperature dependence of the device as was derived from results shown in Figure 8.

Figure 8 shows the influence of temperature in a plastic optical fibre (POF) for different fibre status. Five tests were made: “jacketed POF” (fibre with no action taken on it), “bare POF” (stripped fibre), “polished POF” (fibre whose core was partially polished with a polishing depth of ε = 0.23 mm), “bare POF+glue” (stripped fibre with a glue layer over the cladding) and “polished POF+glue” (fibre partially polished with a glue layer on it). Although polishing the fibre core have significant effects when increasing the temperature (with regards to the stripped POF), it can be seen from Figure 8 that output optical power variations are mainly produced by the effect of gluing when considering the same fibre status, obtaining deviations of 0.15 dB between glued/not-glued bare fibre and 0.12 dB between glued/not-glued polished fibre.

## 5. Conclusions

A novel self-made POF self-referencing intensity-based sensor device for liquid detection has been manufactured and characterized. The sensing area has been obtained by a 3 step process: introducing bending losses, eliminating the fiber cladding and polishing a defined fraction of the fiber core. This technique increases the external media refractive index sensitivity of the sensor (highest sensitivity was obtained for a bending radius of R = 25 mm). Experimental results for the most usual liquids found in industry, like water and oil, were measured. The coupling ratio *K* of the sensor, with increments of ΔK = 0.018 (from air to water), ΔK = 0.060 (from air to oil) and ΔK = 0.042 (from water to oil), allows to distinguish between both the presence and type of the external liquid. Measurement variations were, in all cases, at least one order of magnitude below the average value of the coupling ratio. Measurements also showed a low temperature dependence of *K*, below 1% from its nominal value.

The self-referencing technique was also validated by showing the robustness of the sensor against undesirable losses at any point of the link, because the power ratio as well as the coupling ratio between both output ports of the sensor remains constant.
